# The Prevalence and Implications of Olfactory/Gustatory Dysfunctions among Adult COVID-19 Patients: A Retrospective Cohort Multiethnic Populations Study

**DOI:** 10.3390/tropicalmed7070115

**Published:** 2022-06-23

**Authors:** Wael Hafez, Mahmoud Abdelshakor, Muneir Gador, Ikram Abdelli, Shougyat Ahmed

**Affiliations:** 1 NMC Royal Hospital, 16th Street, Khalifa City P.O. Box 35233, Abu Dhabi, United Arab Emirates; mohamed.mahmoud@nmc.ae (M.A.); muneir.gador@nmc.ae (M.G.); ikram.abdelli@nmc.ae (I.A.); shougyat.ahmed@nmc.ae (S.A.); 2 The Medical Research Division, Department of Internal Medicine, The National Research Center, El Buhouth Street, Ad Doqi, Cairo 12622, Egypt

**Keywords:** olfactory/gustatory dysfunction, COVID-19, severity, ethnicity, UAE

## Abstract

(1) Background: Olfactory and gustatory dysfunctions (OGDs) was found in COVID-19 patients. Only a few studies looked into the prevalence of OGDs in the United Arab Emirates (UAE). The purpose of this study was to estimate the prevalence OGDs among multiethnic COVID-19 patients in the UAE, and its association to patients’ characteristics and disease outcomes; (2) Methods: There were 1785 COVID-19 patients included in our cohort; (3) Results: Males made up most of the study participants (86.3%). A total of 11.7% of the participants reported OGDs. Female gender and ethnicity had significantly higher symptom prevalence (*p* < 0.001). COVID-19 severity had a strong inverse association with OGDs (*p* = 0.007). Other illness outcomes, such as ICU admission, pneumonia development, and mortality, showed no correlation. Males, Asians, and patients with comorbidities all had statistically significantly lower prevalence odds. On the other hand, Emirati, Arab, and Iranian patients had a higher prevalence. COVID-19 patients with OGDs had a considerably shorter time until viral clearance than those without the symptom; (4) Conclusions: in nonsevere COVID-19, olfactory/gustatory dysfunction is common. As a result, it could be applied as a predictive sign for early disease diagnosis and prognosis.

## 1. Introduction

Coronavirus Disease (COVID-19) first appeared in Wuhan, China, in December 2019.

It is caused by severe acute respiratory syndrome coronavirus 2 (SARS-CoV-2) [1]. COVID-19 is a mild to severe infectious disease with dry cough, fever, dyspnea, headache, and gastrointestinal symptoms such as nausea, vomiting, diarrhea, and abdominal discomfort [2].

COVID-19 patients were also shown to have an olfactory and gustatory impairment.

It may serve as an early indicator of mild or moderate COVID-19. In COVID-19, the exact pathogenic mechanism of OGDs is still unknown [3].

In an early Chinese report, just 5.6% of COVID-19 patients experienced a loss of taste [4]. Unlike Giacomelli et al., who found that 33.9% of patients had either olfactory or gustatory dysfunction, and 18.6% had both, symptoms were more common in females (52.6%) than males (25%), and 98% of patients regained their usual sense within 28 days [5].

Olfactory dysfunction was common in a multicenter European investigation (85.6%). Gustatory impairment was very common (88.8%) [6]. The prevalence of olfactory dysfunction was 68% in a study from the United States of America (USA), while gustatory dysfunction was 71% [7].

According to Vaira et al., 80% of COVID-19 patients developed olfactory/gustatory dysfunction after two days of commencement of fever, with symptoms subsiding in 67.5% of patients within a few days. Those who had olfactory/gustatory impairment for more than 10 days had a 2.4 higher risk of severe illness outcomes [8].

Furthermore, 64.4% of patients in Italy had olfactory/gustatory dysfunction, 3% just had taste and smell changes, 11.9% had it before developing other symptoms, 22.8% had it with other symptoms, and 26.7% had it after other symptoms [9].

There is variable presentation of olfactory/gustatory dysfunction, including total or partial loss of smell (anosmia or hyposmia) and taste (ageusia or hypogeusia), altered perception of smell/taste (parosmia and parageusia), and perception of an odor or a taste without any concurrent stimulus (phantosmia and phantogeusia) [10]. Qualitative disturbance of olfactory/gustatory function was also reported in 35.3% of COVID-19 patients, phantosmia and parosmia were reported in 11.8% and 23.5% of patients, respectively, while phantogeusia and parageusia were reported in 17.6% and 23.5% of patients, respectively. Patients also reported a persistent decrease in olfactory but not gustatory dysfunction [11].

Another study of 75 patients self-reporting the degrees of olfactory/gustatory dysfunction indicated that 100% of them had lost their taste, while the loss of sensation was characterized as mild (24%), moderate (13%), and severe (13%). Before being admitted to the hospital, most of them had developed olfactory/gustatory impairment, and the average recovery duration was 17 days [12].

In a comparative study, researchers discovered that patients with self-reported olfactory/gustatory impairment had greater viral load as evaluated by reverse transcription-polymerase chain reaction (RT-PCR) [13]. Another observational cohort study found that female gender, mild illness course, absence of comorbidities, and young age were related to olfactory/gustatory impairment [14]. According to Galluzzi et al., smoking is a risk factor for olfactory dysfunction, and respiratory system allergic illnesses are a risk factor for both olfactory and gustatory dysfunction, while hospitalization and getting supportive respiratory care have the opposite effect [15].

Few reports with modest sample sizes have looked into the prevalence of olfactory/gustatory dysfunction in the UAE [16,17].

This study aimed to determine the prevalence of olfactory/gustatory dysfunction in COVID-19 patients and the relationship between olfactory/gustatory dysfunction and demographics, disease presentation, severity, and outcomes.

## 2. Materials and Methods

### 2.1. Study Design and Participants

This study was a noninterventional retrospective study of COVID-19 patients’ medical records. The study was conducted in the NMC Royal Hospital, Khalifa City, Abu Dhabi, UAE between 8 April 2020 and the end of July 2020.

COVID-19 diagnosis was confirmed by RT-PCR assay—Solgent’s 2019-nCoV RT-PCR Kit—using nasopharyngeal swabs under aseptic operation. Included patients were 1785 adult COVID-19 patients aged 18 or above with different disease severity grades.

Patients Identifiers were removed during the data collection process, with complete protection of patients’ privacy. This study was conducted based on the Declaration of Helsinki. The study was reviewed and approved by the Abu Dhabi Health Research and Technology Ethics Committee, Department of Health, Abu Dhabi, UAE (*Ref:* DOH/CVDC/2021/1330).

### 2.2. Data Collection

Demographic, clinical, and biochemical data were retrospectively analyzed by examining the information from the hospital system INSTA, including gender, age, ethnicity, coexisting diseases, clinical symptoms, and disease outcomes of all patients were extracted from the electronic medical records.

The olfactory/gustatory dysfunction data was collected from the INSTA system in every patient sheet data documented in the present illness symptoms by physicians who clinically assessed the patient in the COVID-19 clinic. The olfactory/gustatory dysfunction was reported as self-reported by the patients. Patients were called by phone to inquire about the olfactory/gustatory dysfunction symptoms in case of missing data.

According to the clinical assessment, all patients had a chest X-ray and/or chest CT on presentation and during follow-up within different interval times. The time interval between the first positive and the first negative PCR test for two consecutive negatives was defined as the time until viral clearance.

### 2.3. Statistical Analysis

After data collection and verification, all data were entered for statistical analysis using R Software version 3.5.2 (20 December 2018), “Eggshell Igloo”, and the appropriate statistical tests were carried out. Quantitative data with a normal distribution were presented as mean ± standard deviation (SD) and range; when normal distribution was violated, data were presented as median and interquartile range. Qualitative data were presented as frequency (n) and percentage (%). Comparative analysis between patients with and without olfactory/gustatory dysfunction was performed and assessed as appropriate by Chi-square test or Fisher exact test. The logistic regression model was used to determine the unadjusted and the adjusted association of olfactory/gustatory dysfunction with patients’ clinical and demographic characteristics and disease outcomes using significant variables from the univariate analysis. The association between olfactory/gustatory dysfunction and the time until viral clearance was conducted using Kaplan–Meier, and the viral clearance rate was estimated using Cox proportional hazards model. The confidence interval was 95%, and the margin of error accepted was 5%. Thus, the *p*-value > 0.05 was considered as nonsignificant and *p* < 0.05 was considered as significant.

## 3. Results

### 3.1. Sociodemographic and Clinical Characteristics of Participants

The study included 1785 COVID-19 patients treated at the NMC Royal hospital. Olfactory/gustatory dysfunction was reported in 11.7% of the study population. Olfactory/gustatory dysfunctions was reported in 12.9%, 12.5%, 11.4%, 5.8%, and 8.6% in patients aged 18 to 29, 30 to 39, 40 to 49, 50 to 59, and those aged more than 60 years old, respectively. There was no statistically significant difference between different age groups. The majority of the study participants (86.3%) were males. However, females showed a statistically significant increased prevalence of olfactory/gustatory dysfunctions vs. males (*p* < 0.001). Race and ethnicity also showed a statistically significant difference regarding olfactory/gustatory dysfunction prevalence. Only 2.4% of South Asian and 14% of East Asian nationalities had developed olfactory/gustatory dysfunction, which was significantly lower than other races (*p* < 0.001). Comorbidities, hypertension, or diabetes had a significant inverse association with olfactory/gustatory dysfunction (*p* = 0.011 and *p* = 0.02, respectively). Only 8.2% of asymptomatic patients developed olfactory/gustatory dysfunction, significantly lower than those with gastrointestinal (GIT) or upper respiratory tract infection (URTI) symptoms (*p* < 0.001). Moreover, the severity of COVID-19 showed a significant inverse association with the development of olfactory/gustatory dysfunction (*p* = 0.007), while no association was observed with other disease outcomes, including intensive care unit (ICU) admission, development of pneumonia, or mortality (Table 1).

### 3.2. Logistics Regression Analysis of the Association between Olfactory/Gustatory Dysfunction and Characteristics of Patients

The odds of olfactory/gustatory dysfunction were 64% lower among those aged 50–59 than those aged < 29 years (OR = 0.36, 95%CI: (0.12–0.96), *p* = 0.050). The odds of olfactory/gustatory dysfunction decreased by 71% among males (OR = 0.29, 95%CI: (0.18–0.47), *p* < 0.001). The odds of olfactory/gustatory dysfunction were significantly lower among East Asians and South Asians (83%, and 97%, respectively) than Africans ((OR = 0.17, 95%CI: (0.07–0.36), *p* < 0.001), (OR = 0.03, 95%CI: (0.02–0.06), *p* < 0.001), respectively).

In opposition, the odds of olfactory/gustatory dysfunction were 4.6 and 2-fold higher among Emirati, Arabs, and Iranian than Africans ((OR = 4.60, 95%CI: (2.09–10.55), *p* < 0.001) (OR = 2.15, 95%CI: (1.08–4.31), *p* = 0.030, respectively). Moreover, the odds of olfactory/gustatory dysfunction were 4.8 and 12-fold higher among patients with URTI symptoms and both URTI and GIT symptoms together than in asymptomatic patients (OR = 4.80, 95%CI: (3.06–7.66), *p* < 0.001), (OR = 12.39, 95%CI: (3.41–43.54), *p* < 0.001, respectively) (Table 2).

### 3.3. Time until Viral Clearance and Olfactory/Gustatory Dysfunction

**Figure 1:**
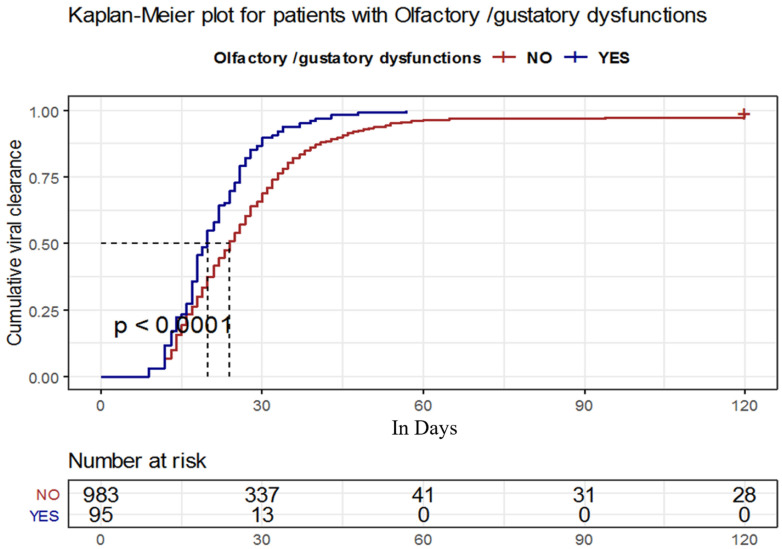
Kaplan–Meier plot for the time until viral clearance among patients with and without olfactory/gustatory dysfunction.

COVID-19 patients who developed olfactory/gustatory dysfunction had a median time until viral clearance = 20 with 95%CI: (18–22) days, which was significantly shorter than those who did not develop the symptom with a median time = 24 with 95%CI: (23–25) days, *p* < 0.001, log-rank = 24 (Table 3) (Figure 1).

**Figure 2:**
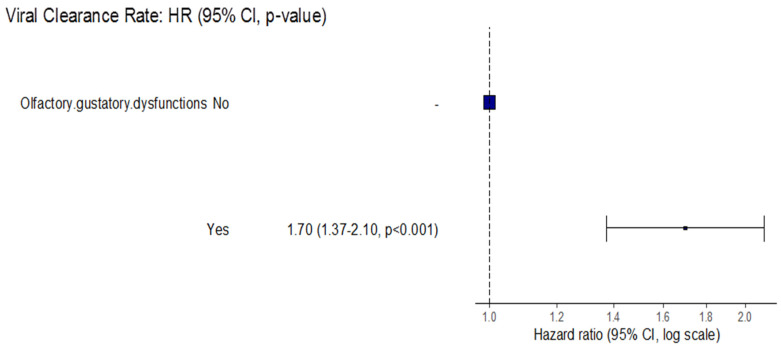
Forest plot for the association between viral clearance rate and olfactory/gustatory dysfunction.

The Cox regression model showed that the viral clearance rate increased significantly among COVID-19 patients who developed olfactory/gustatory dysfunction by 70% compared with those who did not (RR = 1.70, 95%CI: (1.37–2.10, *p* < 0.001) (Figure 2).

## 4. Discussion

This study aimed to study the prevalence of olfactory/gustatory dysfunction in relation to patient features and disease outcomes. Nonsevere COVID-19 individuals showed a considerably higher prevalence of olfactory/gustatory dysfunction. Males, South and East Asians, and patients with comorbidities all revealed a statistically significantly lower prevalence. However, patients with URTI and GIT symptoms and Emirati, Arab, or Iranian patients had a higher prevalence. Our study’s prevalence of olfactory/gustatory dysfunction was 11.7%, which was lower than the findings of Al-Rawi et al. of a 44% loss of smell and a 43% loss of taste in their UAE sample population [16].

In our study, however, both symptoms were assessed simultaneously. Al-Rawi and his colleagues also discovered that non-Arab Asians had the smallest reduction in these sensations, which was similar our findings. Another study by Samaranayake et al. found that anosmia and dysgeusia were prevalent in 9% of mild cases, 6% of moderate cases, and 81% of severe cases [18].

While there was an inverse association between illness severity and OGDs in our study, in comparison to our investigation, the sample size of Samaranayake et al. was smaller; therefore, the prevalence could be underestimated.

Several major systematic reviews and meta-analyses have found that loss of smell is a good predictor of COVID-19 severity [19,20]. Moreover, multiple observational studies have revealed that loss of taste and/or smell is a prevalent symptom among mild-to-moderate COVID-19 patients, implying that olfactory/gustatory dysfunction could be a useful screening tool for early case identification and isolation [6,7,21].

There is a conflicting association between patient demographics and olfactory/gustatory dysfunction. According to Lechien et al., mild COVID-19 individuals, females, and hypertensive patients have a higher prevalence of olfactory and gustatory impairment, consistent with our findings. However, the authors found a positive correlation with diabetes, contradicting our findings [22]. We also discovered no relation between age and the onset of symptoms, which is consistent with Polat et al. [23]. Other studies showed different findings in terms of age, gender, and the presence of comorbidities [6,24,25].

The gender-related difference in host response and disease severity could be attributed to several hormonal, genetic, and inflammatory factors. Lower incidences of severe COVID-19 were reported among females, explaining the high prevalence of olfactory and gustatory dysfunction among them [26,27].

The cause of the inverse association between COVID-19 severity and olfactory and gustatory impairment is unknown. However, various theories have been speculated to explain it. The local inflammatory response and increased olfactory cleft width and volume, or olfactory cleft edema, cause a more rapid immune response and access to the olfactory bulb and neuroepithelium, resulting in injury or degeneration [28,29,30,31,32]. SARS-CoV-2 also disrupts the function of the olfactory epithelium by destroying sustentacular cells and Bowman cells, causing changes in taste and smell sensations, according to recent experimental research [33,34]. Anosmia could also result from the olfactory bulb atrophy based on the magnetic resonance imaging (MRI) and CT findings in COVID-19 patients, as shown in several studies [32,35,36].

Different brain abnormalities due to SARS-CoV-2 infection were also reported, including the reduction in grey matter, cognitive decline, the global reduction in the brain size, and tissue damage in the areas that are functionally connected to the primary olfactory cortex [37]. Other COVID-19-related neurological manifestations could result from SARS-CoV-2 invasion to the central nervous system, and include headache, stroke, confusion, depression, encephalitis, and adrenal insufficiency [38,39]. Persistence of neurological symptoms after recovery was also reported, such as depression, anxiety, poor concentration, anosmia, and memory loss [40].

Another probable mechanism is the virus attaching to the ACE-2 receptors, which are extensively expressed in the nasal mucosa and tongue. The resulting inflammatory response directly affects the olfactory and gustatory receptors [34,41,42].

Cazzolla et al. showed a simultaneous increase in the proinflammatory cytokine Interleukin-6 (IL-6) with the loss of smell and taste among COVID-19 patients [43]. Locatello et al., on the other hand, found that increased IL-10, an anti-inflammatory cytokine, on admission could be a good predictor of recovery from olfactory/gustatory impairment [44].

These findings underline the importance of the inflammatory state in COVID-19 patients’ admission, development, and olfactory/gustatory dysfunction progression. Other possibilities include the occupancy of sialic acid receptors and taste particle destruction. [45]. Other authors stated that the loss of taste is caused by a reduction in the contribution of scent to the perception of distinct flavors [46].

A prior study found that having olfactory/gustatory impairment was associated with a five-fold lower chance of death. As a result, the existence of this symptom would indicate a favorable COVID-19 prognosis [47].

In our study, patients with concurrent URTI or GIT symptoms had higher olfactory/gustatory impairment than asymptomatic COVID-19 patients. Previous research has shown that olfactory and gustatory impairment can occur alone or in combination with other COVID-19 symptoms [48,49]. Inciarte et al. demonstrated a significant association between olfactory and gustatory dysfunction and the development of additional symptoms such as cough and hyporexia [50]. While numerous studies have found no link between olfactory/gustatory dysfunction and rhinorrhea or nasal blockage development, others have reported a possible association [24,51]. This could be explained by the higher expression of ACE-2 in the GIT than the lung, and also the increased occurrence of URTI or GIT symptoms in patients with OGDs than in asymptomatic patients may indicate the higher expression of ACE-2 in symptomatic patients than asymptomatic ones, or the presence of the ACE-2 polymorphism that may impact the course of COVID-19 [52,53].

Olfactory dysfunction developed before other symptoms in 11.8% of mild-to-moderate COVID-19 patients, according to Lechien et al. [6], highlighting the importance of olfactory dysfunction as an early detection marker for SARS-CoV-2 infection. However, during the course of the COVID-19 disease, olfactory, gustatory, or both dysfunctions can arise [54].

Here, olfactory/gustatory dysfunction was significantly low among COVID-19 patients from East Asia and South Asia. At the same time, it was significantly higher among Emiratis, Arabs, and Iranians, which is similar to the previous studies from countries in South and East Asia [4,55,56,57]. The ethnic differences could be attributed to ACE-2 variants that vary in their frequency, different SARS-CoV-2 genotypes and variants, or variable pathogenic susceptibility of populations to SARS-CoV-2 infection or disease outcomes [56,58].

We revealed that patients who had olfactory/gustatory impairment had a considerably shorter time until viral clearance. Taziki Balajelini et al. observed greater CT values and lower viral load in individuals with olfactory or gustatory impairment, which is consistent with our findings [59]. In opposition to our observation, several studies reported lower CT values and a long time until viral clearance and the recovery of olfactory/gustatory disorders [13,60], while Cho et al. found no association between the CT value and olfactory/gustatory impairment degree or recovery duration [61]. These contradictory findings call for more research into the association between viral load and the development of olfactory/gustatory dysfunction for a better prognosis of the recovery of these symptoms and predicting COVID-19 outcomes.

Our study has several limitations: subjective self-reported assessment rather than objective taste and smell measurement, probable recall bias, and lack of chemosensory impairment grading. As a result, underreporting of the symptom cannot be ruled out. Furthermore, no relevant pharmaceutical or rehabilitative intervention that could have altered the evolution of olfactory and gustatory impairment during the study period was documented. However, in a clinical approach that considers the quality of life, an objective evaluation of the disorders may be more important than a subjective assessment. Additionally, because there was no control group, the general population’s prevalence of chemosensory dysfunction could have skewed our findings. Another limitation is that our study was conducted between April and July 2020, and there was only the wild type of SARS-CoV-2 present, with no Delta or Omicron, so no dates regarding these strains were included.

## 5. Conclusions

In conclusion, olfactory/gustatory dysfunction is frequent in nonsevere COVID-19 patients. Gender, ethnicity, the development of respiratory of GIT symptoms, and COVID-19 severity all are significantly associated with developing this symptom. In addition, the time until viral clearance was considerably shorter among those patients who experienced olfactory/gustatory dysfunction. Further research into the pathophysiological effect of various patient characteristics on the development of olfactory and/or gustatory impairment, as well as its predictive usefulness, is recommended.

## Figures and Tables

**Table 1:** Comparative analysis between patients with and without olfactory/gustatory dysfunction regarding demographics, presentation, disease severity, ICU admission, and mortality.

Independent Variables	Total N = 1785	Olfactory/Gustatory Dysfunctions		*p* Value
		No 1576 (88.3%)	Yes 209 (11.7%)	
**Demographics**				
Age (years)	18–29	357 (87.1)	53 (12.9)	0.136
	30–39	676 (87.5)	97 (12.5)	
	40–49	366 (88.6)	47 (11.4)	
	50–59	145 (94.2)	9 (5.8)	
	60+	32 (91.4)	3 (8.6)	
Gender	Female	164 (67.2)	80 (32.8)	<0.001
	Male	1412 (91.6)	129 (8.4)	
Race	Africans	80 (59.3)	55 (40.7)	<0.001
	Arab and Iranian	33 (47.8)	36 (52.2)	
	East Asians	80 (86.0)	13 (14.0)	
	Emirati	19 (31.7)	41 (68.3)	
	South Asians	1317 (97.6)	32 (2.4)	
	Westerners	47 (59.5)	32 (40.5)	
Comorbidities				
HTN	No	1535 (88.0)	209 (12.0)	0.011
	Yes	41 (100.0)	0 (0.0)	
DM	No	1528 (88.0)	209 (12.0)	0.02
	Yes	48 (100.0)	0 (0.0)	
CVD/CKD	No	1563 (88.2)	209 (11.8)	0.385
	Yes	13 (100.0)	0 (0.0)	
Disease presentation				
Pneumonia	No	951 (88.2)	127 (11.8)	0.52
	Yes	547 (89.4)	65 (10.6)	
Clinical presentation	Asymptomatic	1090 (91.8)	97 (8.2)	<0.001
	GIT	7 (87.5)	1 (12.5)	
	URTI	398 (80.9)	94 (19.1)	
	URTI and GIT	76 (89.4)	9 (10.6)	
Disease severity	Nonsevere	1514 (87.9)	209 (12.1)	0.007
	Severe	62 (100.0)	0 (0.0)	
ICU admission	No	1553 (88.1)	209 (11.9)	0.099
	Yes	23 (100.0)	0 (0.0)	
Mortality	Died	14 (100.0)	0 (0.0)	0.394
	Improved	1562 (88.2)	209 (11.8)	

HTN: Hypertension; DM: Diabetes Mellitus; CVS/CKD: Cardiovascular Disease/Chronic Kidney Disease; ICU: Intensive Care Unit.

**Table 2:** Logistic regression analysis of the association between olfactory/gustatory dysfunctions and patients’ demographics, presentation, severity, ICU admission and mortality (odds ratios).

Risk Factors		Unadjusted OR (95%CI)	Adjusted OR (95%CI)
Age (years)	18–29	Ref.	Ref.
	30–39	0.97 (0.68–1.39, *p* = 0.852)	1.29 (0.77–2.19, *p* = 0.332)
	40–49	0.86 (0.57–1.31, *p* = 0.497)	0.89 (0.48–1.64, *p* = 0.711)
	50–59	0.42 (0.19–0.83, *p* = 0.020)	0.36 (0.12–0.96, *p* = 0.050)
	60+	0.63 (0.15–1.85, *p* = 0.460)	0.26 (0.04–1.26, *p* = 0.118)
Gender	Female	Ref.	Ref.
	Male	0.19 (0.14–0.26, *p* < 0.001)	0.29 (0.18–0.47, *p* < 0.001)
Race	Africans	Ref.	Ref.
	Arab and Iranian	1.59 (0.89–2.86, *p* = 0.121)	2.15 (1.08–4.31, *p* = 0.030)
	East Asians	0.24 (0.12–0.45, *p* < 0.001)	0.17 (0.07–0.36, *p* < 0.001)
	Emirati	3.14 (1.67–6.07, *p* < 0.001)	4.60 (2.09–10.55, *p* < 0.001)
	South Asians	0.04 (0.02–0.06, *p* < 0.001)	0.03 (0.02–0.06, *p* < 0.001)
	Westerners	0.99 (0.56–1.74, *p* = 0.973)	1.32 (0.64–2.77, *p* = 0.453)
HTN	No	Ref.	Ref.
	Yes	0.00 (0.00–7.74, *p* = 0.969)	0.00 (NA-Inf, *p* = 0.991)
DM	No	Ref.	Ref.
	Yes	0.00 (0.00–0.94, *p* = 0.966)	0.00 (NA-Inf, *p* = 0.990)
CVD/CKD	No	Ref.	Ref.
	Yes	0.00 (NA-1714991.48, *p* = 0.973)	0.00 (NA-Inf, *p* = 0.995)
Pneumonia	No	Ref.	Ref.
	Yes	0.89 (0.65–1.22, *p* = 0.470)	1.08 (0.68–1.71, *p* = 0.739)
Clinical presentation	Asymptomatic	Ref.	Ref.
	GIT	1.61 (0.09–9.15, *p* = 0.660)	1.06 (0.04–10.86, *p* = 0.968)
	URTI	2.65 (1.95–3.61, *p* < 0.001)	4.80 (3.06–7.66, *p* < 0.001)
	URTI and GIT	1.33 (0.61–2.61, *p* = 0.438)	12.39 (3.41–43.54, *p* < 0.001)
Disease severity	Nonsevere	Ref.	Ref.
	Severe	0.00 (0.00–40.01, *p* = 0.975)	0.00 (NA-Inf, *p* = 0.987)
ICU admission	No	Ref.	Ref.
	Yes	0.00 (NA-437155.47, *p* = 0.977)	0.00 (NA-Inf, *p* = 0.993)
Mortality	Improved	Ref.	-
	Died	0.00 (NA-278302.50, *p* = 0.972)	-

HTN: Hypertension; DM: Diabetes Mellitus; CVD/CKD: Cardiovascular Disease/Chronic Kidney Disease; ICU: Intensive Care Unit.

**Table 3:** The association between olfactory/gustatory dysfunction and time until viral clearance.

Olfactory/Gustatory Dysfunctions	Median Time until Viral Clearance (Days)	95%CI	*p*-Value	Log-Rank Test
Yes	20	18–22	<0.001	24
No	24	23–25		

## Data Availability

Data is available upon request from the first and corresponding author.

## References

[1] Zhu N., Zhang D., Wang W., Li X., Yang B., Song J., Zhao X., Huang B., Shi W., Lu R. (2020). A Novel Coronavirus from Patients with Pneumonia in China, 2019. N. Engl. J. Med..

[2] Huang C., Wang Y., Li X., Ren L., Zhao J., Hu Y., Zhang L., Fan G., Xu J., Gu X. (2020). Clinical features of patients infected with 2019 novel coronavirus in Wuhan, China. Lancet.

[3] Finsterer J., Stollberger C. (2020). Causes of hypogeusia/hyposmia in SARS-CoV2 infected patients. J. Med. Virol..

[4] Mao L., Jin H., Wang M., Hu Y., Chen S., He Q., Chang J., Hong C., Zhou Y., Wang D. (2020). Neurologic Manifestations of Hospitalized Patients with Coronavirus Disease 2019 in Wuhan, China. JAMA Neurol..

[5] Giacomelli A., Pezzati L., Conti F., Bernacchia D., Siano M., Oreni L., Rusconi S., Gervasoni C., Ridolfo A.L., Rizzardini G. (2020). Self-reported olfactory and taste disorders in patients with severe acute respiratory coronavirus 2 infection: A cross-sectional study. Clin. Infect. Dis..

[6] Lechien J.R., Chiesa-Estomba C.M., De Siati D.R., Horoi M., Le Bon S.D., Rodriguez A., Dequanter D., Blecic S., El Afia F., Distinguin L. (2020). Olfactory and gustatory dysfunctions as a clinical presentation of mild-to-moderate forms of the coronavirus disease (COVID-19): A multicenter European study. Eur. Arch. Oto-Rhino-Laryngol..

[7] Yan C.H., Faraji F., Prajapati D.P., Boone C.E., DeConde A.S. (2020). Association of chemosensory dysfunction and COVID-19 in patients presenting with influenza-like symptoms. Int. Forum Allergy Rhinol..

[8] Vaira L.A., Salzano G., De Riu G. (2020). The importance of olfactory and gustatory disorders as early symptoms of coronavirus disease (COVID-19). Br. J. Oral Maxillofac. Surg..

[9] Spinato G., Fabbris C., Polesel J., Cazzador D., Borsetto D., Hopkins C., Boscolo-Rizzo P. (2020). Alterations in Smell or Taste in Mildly Symptomatic Outpatients With SARS-CoV-2 Infection. JAMA.

[10] Hummel T., Whitcroft K.L., Andrews P., Altundag A., Cinghi C., Costanzo R.M., Damm M., Frasnelli J., Gudziol H., Gupta N. (2017). Position paper on olfactory dysfunction. Rhinol. Suppl..

[11] Ercoli T., Masala C., Pinna I., Orofino G., Solla P., Rocchi L., Defazio G. (2021). Qualitative smell/taste disorders as sequelae of acute COVID-19. Neurol. Sci..

[12] Paolo G. (2020). Does COVID-19 cause permanent damage to olfactory and gustatory function?. Med. Hypotheses.

[13] Jain A.K.A., Kaur P.J., Singh L.K.M., Suman Das S.P. (2021). Is there a correlation between viral load and olfactory & taste dysfunction in COVID-19 patients?. Am. J. Otolaryngol. Neck Med. Surg..

[14] Parente-Arias P., Barreira-Fernandez P., Quintana-Sanjuas B.P.-C.A. (2021). Recovery rate and factors associated with smell and taste disruption in patients with coronavirus disease 2019. Am. J. Otolaryngol. Neck Med. Surg..

[15] Galluzzi F., Rossi V., Bosetti C., Garavello W. (2021). Risk Factors for Olfactory and Gustatory Dysfunctions in Patients with SARS-CoV-2 Infection. Neuroepidemiology.

[16] Al-Rawi N.H., Sammouda A.R., AlRahin E.A., Ali F.A.A., Arayedh G.S.A., Daryanavard H.A., Saeed M.H., Nuaimi A.S.A. (2022). Prevalence of Anosmia or Ageusia in Patients with COVID-19 among United Arab Emirates Population. Int. Dent. J..

[17] AlHamadani E., Zia S., AlRahma A., AlNajjar F. (2021). Anosmia as a Screening Tool for COVID-19 Infection: A Prospective Cohort Study. Dubai Med. J..

[18] Samaranayake L.P., Fakhruddin K.S., Mohammad O.E., Panduwawala C., Bandara N., Ngo H.C. (2022). Attributes of dysgeusia and anosmia of coronavirus disease 2019 (COVID-19) in hospitalized patients. Oral Dis..

[19] Aziz M., Goyal H., Haghbin H., Lee-Smith W.M., Gajendran M., Perisetti A. (2021). The Association of “Loss of Smell” to COVID-19: A Systematic Review and Meta-Analysis. Am. J. Med. Sci..

[20] Purja S., Shin H., Lee J.Y., Kim E.Y. (2021). Is loss of smell an early predictor of COVID-19 severity: A systematic review and meta-analysis. Arch. Pharm. Res..

[21] Lee Y., Min P., Lee S., Kim S.W. (2020). Prevalence and Duration of Acute Loss of Smell or Taste in COVID-19 Patients. J. Korean Med. Sci..

[22] Lechien J.R., Chiesa-Estomba C.M., Varia L.A., De Riu G., Cammaroto G., Chekkoury-Idrissi Y., Circiu M., Distinguin L., Journe F., de Terwangne C. (2021). Epidemiological, otolaryngological, olfactory and gustatory outcomes according to the severity of COVID-19: A study of 2579 patients. Eur. Arch. Oto-Rhino-Laryngol..

[23] Polat B., Yilmaz N.H., Altin G., Atakcan Z., Mert A. (2021). Olfactory and Gustatory Dysfunctions in COVID-19 Patients: From a Different Perspective. J. Craniofac. Surg..

[24] Speth M.M., Singer-Cornelius T., Oberle M., Gengler I., Brockmeier S.J., Sedaghat A.R. (2020). Olfactory Dysfunction and Sinonasal Symptomatology in COVID-19: Prevalence, Severity, Timing, and Associated Characteristics. Otolaryngol. Head Neck Surg..

[25] Moein S.T., Hashemian S.M., Mansourafshar B., Khorram-Tousi A., Tabarsi P., Doty R.L. (2020). Smell dysfunction: A biomarker for COVID-19. Int. Forum Allergy Rhinol..

[26] Lechien J.R., Chiesa-Estomba C.M., Place S., Van Laethem Y., Cabaraux P., Mat Q., Huet K., Plzak J., Horoi M., Hans S. (2020). Clinical and epidemiological characteristics of 1420 European patients with mild-to-moderate coronavirus disease 2019. J. Intern. Med..

[27] Chanana N., Palmo T., Sharma K., Kumar R., Graham B.B., Pasha Q. (2020). Sex-derived attributes contributing to SARS-CoV-2 mortality. Am. J. Physiol. Endocrinol. Metab..

[28] Chetrit A., Lechien J.R., Ammar A., Chekkoury-Idrissi Y., Distinguin L., Circiu M., Saussez S., Ballester M.C., Vasse M., Berradja N. (2020). Magnetic resonance imaging of COVID-19 anosmic patients reveals abnormalities of the olfactory bulb: Preliminary prospective study. J. Infect..

[29] Lechien J.R., Michel J., Radulesco T., Chiesa-Estomba C.M., Vaira L.A., De Riu G., Sowerby L., Hopkins C., Saussez S. (2020). Clinical and Radiological Evaluations of COVID-19 Patients with Anosmia: Preliminary Report. Laryngoscope.

[30] Altundag A., Yıldırım D., Tekcan Sanli D.E., Cayonu M., Kandemirli S.G., Sanli A.N., Arici Duz O., Saatci O. (2021). Olfactory Cleft Measurements and COVID-19-Related Anosmia. Otolaryngol. Head Neck Surg..

[31] Sanli D.E.T., Altundag A., Ylldlrlm D., Kandemirli S.G., Sanli A.N. (2022). Comparison of Olfactory Cleft Width and Volumes in Patients with COVID-19 Anosmia and COVID-19 Cases without Anosmia. ORL J. Otorhinolaryngol. Relat. Spec..

[32] Kandemirli S.G., Altundag A., Yildirim D., Tekcan Sanli D.E., Saatci O. (2021). Olfactory Bulb MRI and Paranasal Sinus CT Findings in Persistent COVID-19 Anosmia. Acad. Radiol..

[33] Soler Z.M., Patel Z.M., Turner J.H., Holbrook E.H. (2020). A primer on viral-associated olfactory loss in the era of COVID-19. Int. Forum Allergy Rhinol..

[34] Lima M.H.L.C.D., Cavalcante A.L.B., Leão S.C. (2021). Pathophysiological relationship between COVID-19 and olfactory dysfunction: A systematic review. Braz. J. Otorhinolaryngol..

[35] Hajjij A., Benslima N., Aasfara J., Bensouda H., Mahi M., Benariba F. (2021). An MRI of the Olfactory Tract in a Case of Post-COVID-19 Persistent Anosmia. Integr. J. Med. Sci..

[36] Chiu A., Fischbein N., Wintermark M., Zaharchuk G., Yun P.T., Zeineh M. (2021). COVID-19-induced anosmia associated with olfactory bulb atrophy. Neuroradiology.

[37] Douaud G., Lee S., Alfaro-Almagro F., Arthofer C., Wang C., McCarthy P., Lange F., Andersson J.L.R., Griffanti L., Duff E. (2022). SARS-CoV-2 is associated with changes in brain structure in UK Biobank. Nature.

[38] Iadecola C., Anrather J., Kamel H. (2020). Effects of COVID-19 on the Nervous System. Cell.

[39] Paul T., Ledderose S., Bartsch H., Sun N., Soliman S., Märkl B., Ruf V., Herms J., Stern M., Keppler O.T. (2022). Adrenal tropism of SARS-CoV-2 and adrenal findings in a post-mortem case series of patients with severe fatal COVID-19. Nat. Commun..

[40] Han Q., Zheng B., Daines L., Sheikh A. (2022). Long-Term Sequelae of COVID-19: A Systematic Review and Meta-Analysis of One-Year Follow-Up Studies on Post-COVID Symptoms. Pathogens.

[41] Mahmoud M.M., Abuohashish H.M., Khairy D.A., Bugshan A.S., Khan A.M., Moothedath M.M. (2021). Pathogenesis of dysgeusia in COVID-19 patients: A scoping review. Eur. Rev. Med. Pharmacol. Sci..

[42] Luchiari H.R., Giordano R.J., Sidman R.L., Pasqualini R., Arap W. (2021). Does the RAAS play a role in loss of taste and smell during COVID-19 infections?. Pharm. J..

[43] Cazzolla A.P., Lovero R., Muzio L.L., Testa N.F., Schirinzi A., Palmieri G., Pozzessere P., Procacci V., Comite M.D., Ciavarella D. (2020). Taste and Smell Disorders in COVID-19 Patients: Role of Interleukin-6. ACS Chem. Neurosci..

[44] Locatello L.G., Bruno C., Maggiore G., Cilona M., Orlando P., Fancello G., Piccica M., Vellere I., Lagi F., Trotta M. (2021). The prognostic role of IL-10 in non-severe COVID-19 with chemosensory dysfunction. Cytokine.

[45] Vaira L.A., Salzano G., Fois A.G., Piombino P., De Riu G. (2020). Potential pathogenesis of ageusia and anosmia in COVID-19 patients. Int. Forum Allergy Rhinol..

[46] Fasunla A.J., Thairu Y., Salami H., Ibekwe T.S. (2021). self-reported olfactory, gustatory and otologic dysfunctions among COVID-19 positive adults in Nigeria—A preliminary report. Ann. Ibadan Postgrad. Med..

[47] Porta-Etessam J., Núñez-Gil I.J., González García N., Fernandez-Perez C., Viana-Llamas M.C., Eid C.M., Romero R., Molina M., Uribarri A., Becerra-Muñoz V.M. (2021). COVID-19 anosmia and gustatory symptoms as a prognosis factor: A subanalysis of the HOPE COVID-19 (Health Outcome Predictive Evaluation for COVID-19) registry. Infection.

[48] Gane S.B.C., Kelly C., Hopkins C. (2020). Isolated sudden onset anosmia in COVID-19 infection. A novel syndrome?. Rhinology.

[49] Eliezer M., Hautefort C., Hamel A.L., Verillaud B., Herman P., Houdart E., Eloit C. (2020). Sudden and Complete Olfactory Loss of Function as a Possible Symptom of COVID-19. JAMA Otolaryngol. Head Neck Surg..

[50] Inciarte A., Cardozo C., Chumbita M., Alcubilla P., Torres B., Cordón A.G., Rico V., Aguero D., García-Pouton N., Hernández-Meneses M. (2021). Gustatory and olfactory dysfunctions in hospitalised patients with COVID-19 pneumonia: A prospective study. BMJ Open.

[51] Ghods K., Alaee A. (2022). Olfactory and Taste Disorders in Patients Suffering from COVID-19, a Review of Literature. J. Dent..

[52] Möhlendick B., Schönfelder K., Breuckmann K., Elsner C., Babel N., Balfanz P., Dahl E., Dreher M., Fistera D., Herbstreit F. (2021). ACE2 polymorphism and susceptibility for SARS-CoV-2 infection and severity of COVID-19. Pharm. Genom..

[53] Zhang J., Garrett S., Sun J. (2021). Gastrointestinal symptoms, pathophysiology, and treatment in COVID-19. Genes Dis..

[54] Qiu C., Cui C., Hautefort C., Haehner A., Zhao J., Yao Q., Zeng H., Nisenbaum E.J., Liu L., Zhao Y. (2020). Olfactory and Gustatory Dysfunction as an Early Identifier of COVID-19 in Adults and Children: An International Multicenter Study. Otolaryngol. Head Neck Surg..

[55] Sahoo P.R., Sahu M., Surapaneni P.S., Maiti A., Vankamamidi R., Panda N., Biswal R.N. (2021). Evolution of olfactory and gustatory dysfunctions in COVID-19 patients in India. Eur. Arch. Otorhinolaryngol..

[56] Von Bartheld C.S., Butowt R., Hagen M.M. (2020). Prevalence of Chemosensory Dysfunction in COVID-19 Patients: A Systematic Review and Meta-analysis Reveals Significant Ethnic Differences. ACS Chem. Neurosci..

[57] Komagamine J., Yabuki T. (2020). Initial symptoms of patients with coronavirus disease 2019 in Japan: A descriptive study. J. Gen. Fam. Med..

[58] Kumar A.A., Lee S.W.Y., Lock C., Keong N.C.H. (2021). Geographical Variations in Host Predisposition to COVID-19 Related Anosmia, Ageusia, and Neurological Syndromes. Front. Med..

[59] Taziki Balajelini M.H., Rajabi A., Mohammadi M., Nikoo H.R., Tabarraei A., Mansouri M., Hosseini S.M. (2021). Virus load and incidence of olfactory, gustatory, respiratory, gastrointestinal disorders in COVID-19 patients: A retrospective cohort study. Clin. Otolaryngol..

[60] Nakagawara K., Masaki K., Uwamino Y., Kabata H., Uchida S., Uno S., Asakura T., Funakoshi T., Kanzaki S., Ishii M. (2020). Acute onset olfactory/taste disorders are associated with a high viral burden in mild or asymptomatic SARS-CoV-2 infections. Int. J. Infect. Dis..

[61] Cho R.H.W., To Z.W.H., Yeung Z.W.C., Tso E.Y.K., Fung K.S.C., Chau S.K.Y., Leung E.Y.L., Hui T.S.C., Tsang S.W.C., Kung K.N. (2020). COVID-19 Viral Load in the Severity of and Recovery from Olfactory and Gustatory Dysfunction. Laryngoscope.

